# Remarks on Cholera Spasmodica

**Published:** 1833-01

**Authors:** John Jones

**Affiliations:** Member of the Royal College of Surgeons, London.


### CHOLERA SPASMODICA.

*Remarks on Cholera Spasmodica.* By John Jones, Esq., Member of the Royal College of Surgeons, London.

The general consternation produced by the ravages of this mysterious disease, since its introduction into this country; the various opinions respecting its real nature entertained by medical men; and the no less various and contradictory plans of treatment recommended, which so prominently occupy the pages of the medical periodicals, sufficiently declare the imperfect and unsettled notions which still prevail respecting it, and how inconclusive have hitherto been the principles which have guided the profession in the efforts to check its progress or diminish its fatality. To reconcile these discordant opinions, and to establish a rational treatment on correct physiological and pathological data, is a desideratum of the utmost importance; and every individual who imagines he has fresh views on this momentous subject is not only justified in publishing them, but is bound by a solemn duty to do so, that the profession at large might judge of their value, and how far they are in accordance with established facts and applicable to practice. With these sentiments, and in compliance with the general invitation of the Central Board to the profession to communicate their individual experience in the treatment of Cholera, I venture to add my mite to the accumulated stock of information already obtained.

In the year 1825, several papers of mine were published in the London Medical and Physical Journal, in which I endeavoured to explain the phenomena of many diseases on the long disputed fact of the blood's vitality. The data on which my arguments were founded were, that the blood possesses vitality; that the vital principle, or *pabulum vitæ*, exists principally in arterial blood; that venous blood is comparatively destitute of this principle, which, before it enters the veins, has been expended in supporting the various secretions, and renovating the perpetually wasting energies of the system; that the vital energies become developed through the medium of the brain, spinal marrow, and nerves; and that the operations of the nervous and vascular systems reciprocally depend on each other, the brain and nerves receiving their peculiar vitality from the blood, and the vascular system being enabled to perform its important functions through the agency of nervous influence.

Upon these physiological facts, which I believe incontrovertible, I attempted to show that many diseases are naturally arranged into two great classes; each, however, admitting of modifications, according to the peculiar constitution of the individual, and the exciting and predisposing causes of disease. 1st. Those in which arterial action predominates, which, according to the Brunonian nomenclature, might be called sthenic, and are always accompanied by an augmented and preternatural expenditure of the vital energies. 2. Those of venous congestion, or asthenia, in which these energies are preternaturally depressed.

The accumulated experience we have had of cholera, since its lamented appearance in this country, as well as the testimony of those who have witnessed it abroad, lead to the conclusion that it is a disease emphatically of *congestion*. Postmortem examinations have invariably shown the large veins of the body to be gorged with black blood, which is also found in the heart, and even aorta; and, for the most part, the brain is remarkably vascular, resembling what occurs in those who have died of apoplexy. This state of the venous system co-operating with the mysterious agent by which it is produced, whether contagion or a peculiar state of the atmosphere constitutes epidemica, suddenly diminishes the vital energies; the abdominal veins, which are destitute of valves, and, according to Magendie, possess the function of absorption, become so mechanically distended by congestion, which is rendered more effective by their diminished tone, that, instead of absorbing the fluid contents of the intestines presented to their orifices, and thus, in conjunction with the lacteals, supplying the system with nutrition, they allow the serum of the blood to percolate through their weakened tubes, and, in an awfully augmented degree, add to the depression of the system; the primæ viæ become loaded with this paricidal fluid, which chiefly constitutes the extraordinary evacuations that occur, sometimes consisting of a clear fluid, exactly resembling pale urine, depositing a sediment, at others having the appearance of thin gruel, whey, or rice-water; the colour and sediment of which most probably depend on the admixture of chyle, and perhaps the fibrin of the blood, which, with the serum, has passed through the absorbing veins so strangely deprived of their functions. The abundance and saline properties of the serum escaped into the intestinal canal are alone sufficient to account for the profuse and frequent discharges. These united causes of depression, connected as they are with venous congestion, and the separation from the blood of its saline and watery parts, amply account for the appalling fatality of the disease, its rapid progress, and all its peculiar symptoms, some of which I proceed to consider.

*The reduced temperature and blue colour of the skin.* Animal heat depends principally on respiration, although, from the experiments of Mr. Brodie, which corroborate the opinion of Caverhill, a due development of nervous energy would also appear essential to its production. During respiration, carbonic acid, formed by the union of the oxygen of the atmosphere with the carbon of the blood, is evolved; another portion of oxygen is supposed to be employed in the combustion of hydrogen. These processes, in a state of health, are accompanied by an abundant production of animal heat.

The blood in cholera, deprived, by the escape of its serum, of its usual fluidity and saline constituents, which doubtless are essential to the changes produced by respiration, is prevented from receiving the vivifying influence of oxygen; its carbon is, therefore, retained; the arteries, instead of circulating red blood capable of renovating the wasted energies, contain venous or carbonized blood, which, feebly circulating in the capillaries of the skin, produces its characteristic blueness and icy coldness. To this state of the circulation also, accompanied by an accumulation of blood in the large internal vessels, and a proportionate recession from the surface, may be attributed the extraordinary shrinking of the features, sinking of the eyes, corrugation of the integuments of the fingers, and insensibility of the skin, which will sometimes admit of being pinched or pricked without exciting pain, and so completely is the vital contractility annihilated that, when pinched, the folds remain exactly as on the skin of a corpse.

*Suppression of the Secretions.* This is a remarkable and characteristic symptom: the urinary bladder is generally empty; although bile is found in the gall-bladder, none escapes into the duodenum; the discharges ejected from the stomach and bowels consist principally of serum, and are not the result of secretion, which is defective, and therefore not depending, as usually supposed, on morbid irritability of the mucous membrane.

The process of secretion depending on the healthy state of the capillaries of the secreting organs, is suspended in consequence of the accumulated causes of depression, which so peculiarly mark the disease; and, in addition, the blood being deprived of its serum, from whence the secretions are chiefly derived, these must, of course, be suppressed till this anomalous decomposition of the blood is prevented. We find, accordingly, that the return of the secretions forms the best indication of amendment.

*Spasms.* This symptom seems intimately connected with the diminished energies of the system. It often accompanies the premonitory diarrhœa, and begins with a tingling of the fingers and toes, succeeded by cramps, which gradually pervade the forearms and calves of the legs. In the progress of the disease, the spasms sometimes become dreadful. In a letter I received from my nephew, Mr. Thomas Jones, who was in Paris during the prevalence of cholera in that devoted city, he says "yesterday, as I stood by the bedside of a man during a paroxysm, the effort of the muscles dislocated the lower jaw." The irregular distribution of nervous influence produced by the sudden and intense operation of the depressing causes is sufficient to account for this symptom.

Mechanical pressure on the nerves, by the congestion of contiguous vessels, might also assist in its production.

A remarkable coincidence seems to exist between cholera and the first stage of typhous fever. In both, the vital energies are depressed by the influence of some unknown agent on the nerves, followed by the sudden production of venous congestions of the large vessels of the body and internal organs. In typhus, this state of depression is usually succeeded by increased action of the heart and arteries, constituting febrile reaction. Sometimes, however, it is so great as to prevent this salutary occurrence, when the action of the heart is enfeebled, the circulation tardily performed, pulse weak, heat of the surface below par, the functions of the internal organs imperfectly performed, and the patient suddenly sinks; or, should reaction occur, it is but feebly developed. The vital energies are remarkably depressed through the whole course of the fever, giving a character to its type, and constituting one of its most fatal forms. In cholera, superadded to the above causes of diminished tone, is the escape of serum from the blood, which sufficiently accounts for all its distinguishing phenomena, its rapid progress and awful fatality. In the letter already referred to, my nephew observes, "So sudden is the invasion of the disease, and so rapid its progress, that, in two hours, in some instances, death has seized his prey; and, even whilst taking down a case from the mouth of an individual, it is evident that every moment is plunging him deeper and deeper into that state of profound collapse from which all exertions are vainly tried to rescue him." Those cases in which amendment occurs are frequently followed by fever, which assumes more or less the character of typhus, and is accompanied with a marked state of organic congestions, particularly of the brain. On the extent of these congestions, and the importance of the organs so affected, the malignancy of the consecutive fever seem to depend.

Without entering into a minute detail of the treatment, I shall consider the *modus operandi* of some of the means recommended for combating this fatal disease; many of which are so totally at variance with each other, that, if some have been beneficial, others must have been equally prejudicial.

*Diffusible Stimuli.* These have been profusely administered, with the view of rousing the vital energies and producing reaction. Experience has, however, taught us their inefficiency, and there is too much reason to believe that the fatality of the disease has been in some measure proportioned to the confidence placed in this class of remedies, and the freedom with which they have been employed. Their stimulating effects depend on a due development of nervous energy, and perhaps their absorption into the circulation. In consequence of the torpid state of the nerves, produced by causes over which stimulants can have no influence, and the inverted action of the intestinal veins, by which their function of absorption is suspended, they fail to excite reaction, and, by their constringing effects, they tend to retard the return of the secretions so essential to the recovery of the patient.

*Opium.* To control the morbid irritability of the mucous membrane, erroneously supposed to be the cause of the abundant evacuations, and to relieve the violent spasms which occur, this remedy has been the principal resource of many practitioners, who have considered it their sheet-anchor. As the fluid so copiously evacuated from the stomach and bowels is extraneous, and not the result of increased secretion, although opiates might afford temporary relief, yet, when freely administered, they must retard reaction, and accelerate the fatal progress of the disease, by checking the evacuations and suppressing the secretions. They are only admissible when given in small doses, and conjoined with appropriate aperients.

*Saline Injection into the Veins.* This remedy, founded on the analysis of the blood, which proved it to be deprived of its serum, was intended to supply this deficiency. The bold suggestion of injecting large quantities of a saline fluid into the veins was speedily adopted, and the profession was astounded by the novel fact that such injections could be made, not only with impunity, but even with effects apparently the most beneficial. Unfortunately, however, these effects were but of short duration: although the serous part of the blood was thus renewed, its escape from the absorbing veins was not prevented; and, although reaction was produced, the dreaded collapse returned with as much, if not more, intensity than if the operation had not been performed. It has been fairly tried three times in this town: in each case, the patient, for a short time, seemed revived, and the symptoms generally ameliorated; but collapse speedily recurred, and terminated together the life of the patient and the solicitude of the practitioner.

*Calomel.* This invaluable medicine has been found, by almost universal experience, both in Asia and Europe, to be a remedy most entitled to confidence. It not only excites the secretions generally, but has a peculiar effect in increasing that of the liver in particular; it has also a powerful influence on the absorbent system, and seems to have a direct effect in restoring the absorbing function to the intestinal veins. In those cases of amendment treated with diffusible stimuli, as brandy, ether, &c., and opium, with which it has usually been combined, it has doubtless obviated the evils of such treatment, and has been the principal cause of recovery.

*Aperients.* This class of remedies has not till lately been duly appreciated. Experience has, however, fully established their utility, and they are now considered, by most practitioners, one of their chief remedial resources. They not only favor the evacuation of the serous contents of the intestines, but, by restoring the secretions, relieve the existing congestions, which, by their intensity and *sudden* production, may be considered the principal cause of the inverted action of the absorbing veins. This anomalous state, therefore, aperients have an indirect and powerful tendency to correct, and, in conjunction with calomel, fulfil two of the most important indications in the treatment, the re-establishment of the functions of the secreting organs, and that of the absorbing veins of the intestines. When these most important objects are secured, reaction and convalescence speedily ensue, if the remaining energies are sufficiently great to recover from the shock the system has sustained.

*Bleeding.* From the extreme state of depression which so peculiarly distinguishes the disease, the propriety of bleeding would appear very questionable, if experience had not fully proved its efficacy. If employed sufficiently early, it has a direct tendency to unload the congested vessels, and thereby to restore the absorbing function of the intestinal veins, and consequently prevent the further escape of serum from the blood, by enabling the heart and arteries to act with greater energy; it also equalizes the circulation, increases the secretions, and facilitates reaction. These beneficial results, however, can only be expected when bleeding is employed early; for, should it not relieve the oppression of the system, it must accelerate exhaustion.

*External Warmth, Rubefacients, Sinapisms, &c.* The extraordinary reduction of temperature which accompanies this disease, naturally suggests the propriety of counteracting it by external warmth, &c. From the preceding pathological views, it is evident that such means are wholly insufficient to effect the object intended, and that we can expect warmth to be restored to the surface only by removing the causes of depression on which the coldness depends. These means, therefore, can be considered but of secondary importance, and must be employed only as auxiliaries to those remedies which tend to restrain the escape of serum from the blood, and remove the existing congestions. When these impediments to the due development of nervous influence are removed, the warmth of reaction generally soon follows, and doubtless is facilitated by the conjoint employment of external warmth, stimulating embrocations, sinapisms, &c.

The following cases illustrate the principles of treatment recommended in the preceding remarks.

Sarah Ryley, æt. twenty-nine. I was hastily called to attend this woman, who, the messenger informed me, was affected with cholera. There appeared a general excitement in the neighbourhood, and, on entering the patient's room, which was crowded with women, found her violently retching; her features remarkably shrunk; eyes sunk deep in the sockets, surrounded with a dark-coloured areola; skin of an icy coldness, dirty brown colour, and nails blue; pulse hardly perceptible, and, on seeing her a short time afterwards, quite imperceptible. Retching and purging had been for the last hour almost incessant: little was vomited, but alvine evacuations were passed involuntarily, and so watery and profuse as to soak through the bed, leaving no appearance on the sheet except a few flocculi. Violent pain in the bowels, and severe spasm of upper and lower extremities. There appeared so much apathy of perception as to deprive her of consciousness. There had been no premonitory symptoms, as, till within an hour of my seeing her, she was as well as usual.

I immediately ordered the women, whose curiosity had so strangely overcome their fears, to leave the room, excepting such as were really necessary to attend the patient. Hot bricks were applied to the feet, and a bladder, partly filled with hot water, to the epigastrium; which, together with the palms of the hands and soles of the feet, was to be frequently rubbed with a liniment composed of a watery mixture of mustard and turpentine; and the following pills to be taken every half hour till reaction occurred, and afterwards every hour; and an emulsion of castor oil every two hours, till the evacuations became feculent: R. Submur. Hydr. gr. ij.; Extr. Colocynth. comp. gr. iij.; Pulv. Opii, gr. $\frac{1}{2}$; Ol. Carui, gtt, i. M, fiat pil. ij.

Before the medicines were prepared, the assistant of another practitioner had accidentally called, and, not knowing what I had prescribed, ordered mustard poultices to the calves of the legs.

After the first dose of the pills, the retching ceased, and in a few hours the evacuations became feculent and of a dark colour, accompanied with reaction and general amendment. The following day she had slight fever, which soon subsided, and left her in her usual health.

I was particularly struck with the altered appearance of my patient when she became convalescent. On first seeing her under the influence of the disease, she appeared at least fifty years old; on visiting her the following day, I was surprised to find her a handsome young woman.

Elizabeth Cutts, æt. twenty-four, the wife of a pedlar, with whom she had travelled three weeks before her present illness, and about six weeks ago was delivered of a child, which she is now suckling, since which her health has been very delicate. The day after, having participated in the public reform rejoicings, she was affected with diarrhœa, which continued till the following morning, when I first saw her. During the night, vomiting and purging had been very violent, and were still unabated; the skin remarkably cold; severe cramps of the extremities; pulse hardly perceptible; tongue cold and exsanguinous; had passed little or no urine since the commencement of her illness; alvine evacuations resembled thin gruel mixed with milk.

The same treatment, with the exception of mustard poultices, was pursued as in the former case, and with the same happy result. In a few hours the motions became feculent and dark-coloured, followed by reaction, when about twelve ounces of blood were taken from the arm: the blood was almost black, with scarcely any serum. In the course of three days she was restored to her usual health, without the occurrence of consecutive fever.

Since last spring, cholera has repeatedly appeared in this town, but its prevalence has not been extensive; the number of cases reported being only twenty-seven, out of which there have been fourteen deaths. After having ceased for about a month, it has again appeared, and, within these few days, five cases have occurred, and three deaths. Hitherto it has been invariably isolated, attacking one individual, and not extending to others of the same house or yard; or a solitary individual has been seized, after having taken indigestible food, and sometimes without any assignable cause. For the most part, the persons affected have been of the lowest class, and residing in the most confined and filthy situations, and in the neighbourhood of water, of which there is great abundance in and about the town. There have, however, been three fatal exceptions to this rule; a lady and two gentlemen of great respectability: the former, the wife of a surgeon, had been in a delicate state of health for some time previously, was affected with diarrhœa, and had a great dread of the disease, which was considerably heightened by her husband having been at the post-mortem examination of a child who had died of it in the course of nine hours. This lady's veins were injected, which produced the usual temporary reaction, succeeded by a speedy return of the deadly collapse, which terminated her existence. Great imprudence in diet seemed to be the exciting cause of the disease in the other two, producing diarrhœa, and then the peculiar choleric symptoms.

It has generally been preceded by diarrhœa, which, during the whole period, has prevailed as an epidemic: in some cases, however, it has commenced with collapse, and terminated fatally in the course of a few hours. The diarrhœa, if attended to sufficiently early, has been easily controlled by the usual means. The circumstances which seem to have had a considerable influence in preventing hitherto the spread of the disease are, 1. The measures adopted by the inhabitants in anticipation of its appearance, which, by means of a very liberal public subscription, were upon a large and efficient scale; nuisances were removed; the indigent supplied with blankets and coals, and their houses well whitewashed. 2. The general cleanliness of the town, which is well built, and not over-populated. 3. The facilities afforded to the indigent and working classes for obtaining prompt medical assistance, not only by means of the General Infirmary, but also by the admirable working of a self-supporting Dispensary, established about two years ago on the principles recommended by Mr. Smith, of Southam. Between fourteen and fifteen hundred cases have been treated by the medical officers of this institution during the last year, many of which were diarrhœa, and there is little doubt, without timely aid, would have become genuine spasmodic cholera.

